# IROme, a New High-Throughput Molecular Tool for the Diagnosis of Inherited Retinal Dystrophies

**DOI:** 10.1155/2013/198089

**Published:** 2012-12-26

**Authors:** Daniel F. Schorderet, Alexandra Iouranova, Tatiana Favez, Leila Tiab, Pascal Escher

**Affiliations:** ^1^Institute for Research in Ophthalmology (IRO), Grand-Champsec 64, 1950 Sion, Switzerland; ^2^Department of Ophthalmology, University of Lausanne, 1004 Lausanne, Switzerland; ^3^Ecole Polytechnique Fédérale (EPFL), 1015 Lausanne, Switzerland

## Abstract

The molecular diagnosis of retinal dystrophies is difficult because of the very important number of genes implicated and is rarely helped by genotype-phenotype correlations. This prompted us to develop IROme, a custom designed in solution-based targeted exon capture assay (SeqCap EZ Choice library, Roche NimbleGen) for 60 retinitis pigmentosa-linked genes and three candidate genes (942 exons). Pyrosequencing was performed on a Roche 454 GS Junior benchtop high-throughput sequencing platform. In total, 23 patients affected by retinitis pigmentosa were analyzed. Per patient, 39.6 Mb were generated, and 1111 sequence variants were detected on average, at a median coverage of 17-fold. After data filtering and sequence variant prioritization, disease-causing mutations were identified in *ABCA4*, *CNGB1*, *GUCY2D*, *PROM1*, *PRPF8*, *PRPF31*, *PRPH2*, *RHO*, *RP2*, and *TULP1* for twelve patients (55%), ten mutations having never been reported previously. Potential mutations were identified in 5 additional patients, and in only 6 patients no molecular diagnosis could be established (26%). In conclusion, targeted exon capture and next-generation sequencing are a valuable and efficient approach to identify disease-causing sequence variants in retinal dystrophies.

## 1. Introduction

Retinitis pigmentosa (RP) (MIM number 268000) is a group of genetically highly heterogeneous-inherited retinal dystrophies [1]. Typically, night blindness starts during adolescence, and patients progressively loose the rod photoreceptor-mediated peripheral vision. At later stages, the cone photoreceptors also become affected, constricting vision over time to the most central fovea and eventually resulting in complete blindness. To date, more than fifty genes have been linked to nonsyndromic RP (RetNet; http://www.sph.uth.tmc.edu/RetNet/). Inheritance can be autosomal dominant (AD), autosomal recessive (AR) or X-linked, and, rarely, mitochondrial or digenic [2]. Sporadic or simplex cases account for about 30% [3].

The molecular diagnosis of RP is difficult because (i) there is no genotype/phenotype correlation in a vast majority of patients, (ii) a high intra- and interfamilial variability of clinical phenotypes is observed in patients carrying the same causative mutation, (iii) different mutations in a same disease-linked gene cause highly variable clinical phenotypes if not clinically distinct retinal degenerations, and (iv) overlapping clinical phenotypes and disease-linked genes exist with additional retinal degenerations, that is, early-onset Leber congenital amaurosis (LCA), congenital stationary night blindness (CSNB), cone-rod dystrophies (CRD), enhanced S-cone syndrome (ESCS), or syndromic RP in Bardet-Biedl and Usher syndrome [2]. However, identification of RP-linked sequence variants is important for genetic counseling and patient management.

Similar to other Mendelian disorders, mutations in RP patients were identified until recently by linkage mapping and subsequent Sanger sequencing of candidate genes [4]. For molecular diagnosis, the validated RP mutations could be detected by arrayed primer extension (APEX) chip technology [5]. However, a low success rate in detecting mutations by APEX was inherent to the genetic heterogeneity of RP patients, and in a cohort of 272 Spanish families affected by ARRP, causative mutations were identified in only 11% of them [6].

The development of next-generation sequencing (NGS) tools in recent years has allowed the production of an enormous volume of sequencing data at low costs [7]. Whole genome sequencing and downstream data handling remains cost and labor intensive, limiting its use in routine mutation detection [8]. Targeted capture of the about 30 Mb of protein-coding regions in the human genome, the so-called exome, reduced the sequencing and data handling effort by a factor of 100 and allowed the identification of mutations in unrelated patients affected by the same syndrome [9]. Exome sequencing has since been widely used as a tool for Mendelian disease gene discovery [10, 11]. Initially array-based, targeted sequence capture has become easy-to-use, thanks to the development of in-solution capture methods [12]. Finally, benchtop high-throughput sequencers made exome sequencing available to small-size diagnostic laboratories [13]. 

These technological advances prompted us to develop a custom designed in solution-based targeted capture assay, called IROme, for the detection of mutations located in the exons, including complete 3′-untranslated regions (UTR), intron-exon boundaries and potential promoter, and 5′-UTR regions of 63 genes on a 454 GS Junior sequencing platform.

## 2. Material and Methods

### 2.1. Patients and DNA Samples

These studies were approved by the Swiss Federal Department of Health (authorization number 035.0003-48) and followed the principles of the Declaration of Helsinki. The 23 patients analyzed in this study were of Swiss, Algerian, and Tunisian origin. Blood samples were collected after informed consent. Genomic DNA was extracted from peripheral blood using a Nucleon BACC2 genomic DNA extraction kit (GE Healthcare, Glattbrugg, Switzerland). Four patients had been previously analyzed at Asper Biotech for known RP-linked mutations by APEX technology [5].

### 2.2. Design of Solution-Based Capture Assay for Retinitis Pigmentosa-Linked Genes

Exons of targeted genes were identified in the reference human genome version hg19 (http://www.ensembl.org/) (Table 1). For each exon 50 bp were added in both 5′ and 3′ of the exon, including the complete 3′UTR for each gene. Potential alternative transcripts were also considered in the design. To include potential proximal promoters, an additional 1000 bp in 5′ of the first exon of each gene, containing the complete 5′-UTR, were added. The resulting custom-designed SeqCap EZ Choice library (NimbleGen, Roche) was called IROme, version 1.

### 2.3. GS Junior Sequencing

**Figure 1:**
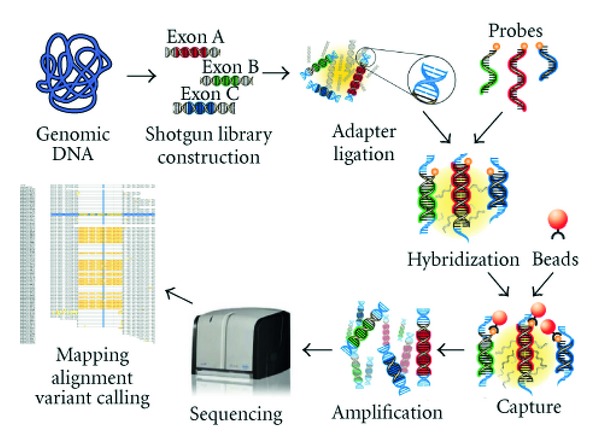
Workflow of the custom-designed targeted exome liquid hybridization capture assay IROme. Genomic DNA from patients was fragmented by nebulization and used for shotgun library construction (454 Roche GS Titanium Rapid Library). Upon adapter ligation, target enrichment is achieved by hybridizing the processed genomic DNA to biotinylated probes (Roche NimbleGen SeqCap EZ Choice). After biotin-streptavidin-based capture and washing, DNA was amplified by emulsion PCR and sequenced on a454 Roche GS Junior Sequencer. Sequencing data was aligned and mapped with the Roche 454 Reference Mapper program. Figure adapted from Roche NimbleGen technical information (http://www.nimblegen.com/products/seqcap/index.html).

The workflow for GS Junior sequencing is summarized in Figure 1. DNA concentrations were measured on a NanoDrop spectrophotometer (Thermo Fisher Scientific, Wilmington, DE). 500 ng of gDNA were fragmented by nebulization, and size selected by Agencourt AMPure XP beads (Beckman-Coulter, Beverly, MA) to obtain fragments between 500 and 1200 bp. Adaptors provided in the GS Titanium Rapid Library Preparation Kit (Roche, Basel, Switzerland) were ligated to the fragmented DNA and then quantified by fluorometry (QuantiFluor, Promega, Madison, WI). This library was amplified by ligation-mediated (LM)-PCR using specific 454 primers. Then, 1 *μ*g of the PCR amplification product was dried down with COT-DNA (Roche) and 454-Hybridization Enhancing Primer in a Speedvac. The pellet was resuspended in NimbleGen's hybridization buffer and hybridized to the custom-designed SeqCap EZ Choice library (NimbleGen, Roche), called IROme v1, for 70 h at 47°C in a thermocycler. The captured DNA was bound to Streptavidin M-270 Beads (Invitrogen Dynal, Oslo, Norway) for 45 min at 47°C and, using a magnet support, washed with the 4 different NimbleGen buffers provided according to the manufacturer's instructions. The captured DNA-Beads were amplified by LM-PCR using the same specific 454 primers as before. Captured and noncaptured DNA was subjected to quantitative PCR on a Lightcycler480II (Roche, Basel, Switzerland) to measure the relative fold enrichment of the targeted sequences. Postcapture samples with an enrichment higher than 200-fold were further processed. According to the 454 GS Junior protocol (Roche), an emulsion PCR was done on 2 molecules per beads. After PCR, the beads were collected, washed, and bound to the Enrichment Beads. The enriched DNA was then eluted and quantified with the provided bead counter. Sequencing was performed following the 454 GS Junior protocol. Briefly, 500′000 enriched DNA beads were mixed with Packing Beads. Then, the PicoTiterPlate (PTP) was sequentially loaded with Prelayer Beads, DNA-Packing Beads, Postlayer Beads, and PPiase Beads. Finally, the PTP was mounted in the 454 GS Junior Sequencer, and the program was run in full processing for shotgun sequencing. 

### 2.4. Data Analysis

**Figure 2:**
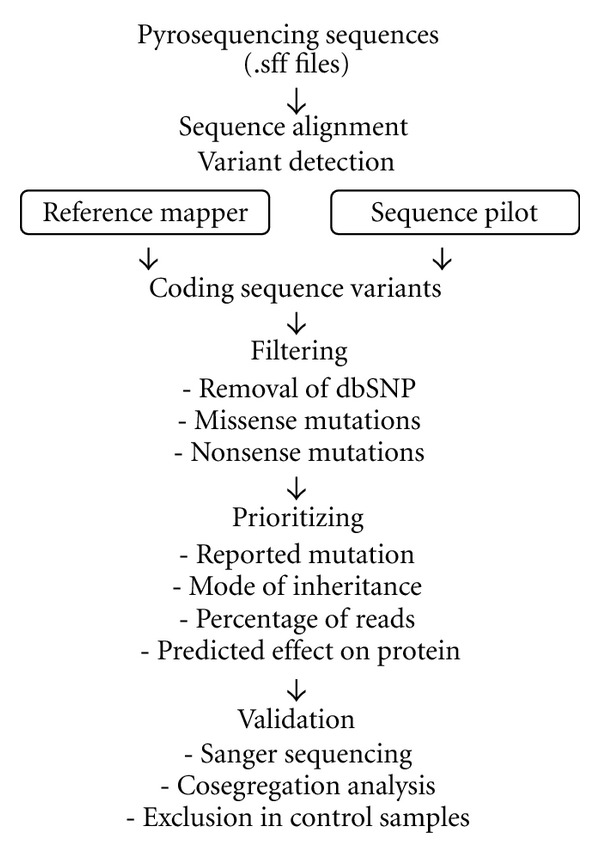
Workflow of data analysis and filtering. The sff (sequence file format) files generated by 454 Roche GS Junior sequencing were imported either into Reference Mapper or Sequence Pilot software. The coding sequence variants were selected from the 454_HCDiffs.txt files that contained all sequence variants. During filtering, coding sequence variants reported in dbSNP were removed, and missense and nonsense mutations kept. The remaining coding sequence variants were prioritized according to known reported mutations, the mode of inheritance, the percentage of sequence reads reporting the variant (threshold of 20%), and the predicted effect on the protein (PolyPhen score).

The workflow for data analysis and data validation is summarized in Figure 2. Sequencing data (.sff file) were analyzed with Roche 454 Reference Mapper program. Reference text (ref.txt) for gene annotations and the snp131 version of the single nucleotide polymorphism database (snp131.txt) were downloaded from the Golden Path database ( http://www.genome.ucsc.edu/). The sequence variants provided by the 454HCDiffs.txt file were filtered for known SNPs (http://www.ensembl.org/Homo_sapiens/Gene/Variation_ Gene/), type of amino acid changes (http://genetics.bwh.harvard.edu/pph2/), and repetitive sequences. An additional in-house developed program was used to check the remaining SNPs against reference sequences obtained in Ensembl. Sequence variants were further prioritized according to inheritance, if family information was available, and to the percentage of reads containing a given sequence variant (threshold at 20%). To analyze the coverage, scripts were written to extract global coverage data from the 454AlignmentInfo.tsv file (unique depth, column 5) and the quality of coverage at each targeted nucleotide (column 4). Part of the sequencing data was analyzed by Sequence Pilot version 3.5 (JSI Medicals, Kippenheim, Germany).

### 2.5. Data Validation

Sanger sequencing validated all potential pathogenic sequence variants. Briefly, 20-bp primers flanking the given region and yielding amplicons of 300–600 bp were designed (primer sequences available on request). The polymerase chain reaction (PCR) was performed in a total volume of 20 *μ*L, containing 20 ng genomic DNA, 1 mM of each primer (Eurogentec, Liège, Belgium), and 10 *μ*L FastStart PCR Master Mix (Roche, Basel, Switzerland). Amplification was performed in a GeneAmp 9700 thermal cycler (Applied Biosystems, Carlsbad, CA, USA) with the following conditions: 1 min at 95°C, 35 cycles of 1 min at 94°C, 1 min at 58°C, 1 min at 72°C, and, a final elongation step at 72°C for 10 min. PCR-amplified products were purified with an Invitek MSB Spin PCRapace kit (STRATEC Molecular GmbH, Berlin, Germany). Sanger sequencing was done in a final reaction volume of 10 *μ*L, using BigDye Terminator v3.1 (Applied Biosystems) with forward and reverse primers. Fragments were separated on an ABI PRISM 3100 genetic analyzer (Applied Biosystems). Sequences were analyzed using Chromas 2.23 software (Technelysium, Tewantin, QLD, Australia).

## 3. Results and Discussion

### 3.1. IROme: Design and Validation of the Assay

The vast genetic heterogeneity of RP prompted us to develop a custom-designed hybridization-based targeted exon capture assay, called IROme. Enrichment was targeted towards a total of 63 genes (942 exons), of which 60 genes were linked to RP, LCA, and related retinal dystrophies (Table 1). The exon ORF15 of *RPGR* was not included in the assay because of the presence of repetitive sequences. Two RP- or LCA-linked genes, *IDH3B* and *RD3*, had been reported only in a single family so far and were not included in this version of IROme. Conversely, two candidate genes that were linked to retinal degeneration in mice, but not humans, were added to the assay (*TUB* and *LPCAT1*). A third candidate gene located on chromosome X, *CNGA2*, was included because of its homology to *CNGA1*. The total of targeted regions spans 394′758 bp.

Of note, after the design of IROme was completed, *TTC8* (*BBS8/RP51*), *C8ORF37,* and *MAK* were linked to RP, and *KCNJ13* and *NMNAT1* to LCA. These latter genes, as well as *IDH3B* and *RD3*, will be included in a future version of IROme.

Patients 1–4 had previously been investigated by APEX technology for known RP-linked mutations [5]. All nucleotides tested by APEX were correctly detected by IROme, with a 98.9% accuracy of the sequence reads for nucleotides at a homozygous state (Table 2). A p.USH2A-V2562A mutation had been detected by APEX in patient 2 in a heterozygous state, and this was correctly validated by IROme (46.8% of the sequence reads at 47-fold coverage).

As an additional control, the IROme assay was tested on genomic DNA of a previously described family of Algerian origin, affected by LCA or early onset retinal degeneration [14]. The causative 6-base in-frame duplication c.* TULP1-*1593_1598dupTTCGCC was readily detected in exon 15 (Table 3, patient 5).

### 3.2. IROme: Variant Detection, Coverage, and Data Filtering

A total of 23 RP patients were analyzed by IROme (Table 3). Pyrosequencing generated an average of 39.6 ± 14.1 Mb per patient, with an average read length of 408 ± 48 bp. These long read lengths are comparable to published analyses, where the Roche 454 GS Junior generated the longest read lengths, in comparison to the other benchtop high-throughput sequencing platforms, MiSeq (Illumina) and Ion Torrent PGM (Life Technologies) [13].

**Figure 3:**
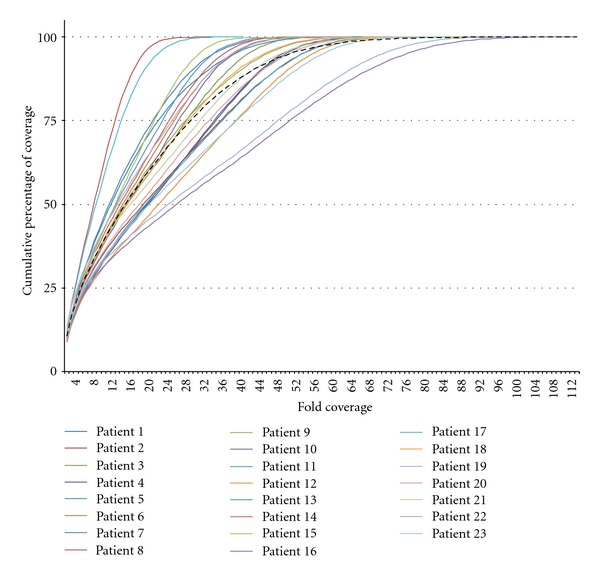
Fold coverage of targeted sequences. For each patient the unique depth data provided by column 5 of the 454_AlignmentInfo.tsv file was used to estimate the coverage per targeted bp. The onefold coverage data corresponding to reference genome sequences used for alignment purposes, but not targeted by IROme, were removed. The coverage data is represented as cumulative percentage; that is, indicating what percentage of targeted bp has a minimal coverage of *x*-fold (*x* axis represents the fold coverage). The average coverage for all patients is represented as a black dashed line, and the median coverage for all patients is 17-fold.

On average per patient, 1′111.7 ± 222.2 sequence variants were found (range: 736–1′826). Among these, 90.1 ± 10.0 were located in coding sequences, and a further 42.1 ± 4.7 were changing the amino acid sequence. By considering all patients, the median coverage was 17-fold, with a maximal 112-fold coverage in one exon of patient 16 (Figure 3). No coverage was observed for four exons (0.3%): exons 1 of *RP9*, *IMPDH1,* and *LPCAT1* and an alternative exon 2 of *CNGA2*. These exons contained GC-rich and/or repetitive sequences impeding efficient probe design and targeting [15]. Another 15 exons were not covered in all patients (1.6%). Because these exons were not restricted to the 5′ regions, absence of coverage was attributed to technical limitations or, as observed for patient 9, to a deletion (see below).

For patients 20 and 21, two potential heterozygote mutations had been detected at 22.6% (53-fold coverage) and 21.3% (61-fold coverage), respectively. However, these two sequence variants could not be validated by Sanger sequencing. For further patient analyses, a more stringent threshold up to 35% of sequence reads might be used for prioritization of sequence variants. Alternatively, a dynamic threshold could be implemented, starting at a high stringency and going down until one or two mutations are identified.

In conclusion, the design of IROme resulted in an over 98% coverage of the targeted exons. The variant detection workflow could be improved by further increasing the quality of the sequencing data, that is, by using a benchtop sequencer less prone to homopolymer-associated insertion/deletion errors (e.g., MiSeq, Illumina) [13] and high-fidelity DNA polymerases [16].

### 3.3. IROme: Molecular Diagnosis on RP Patients

IROme analysis yielded in definite diagnosis for 55% of the RP patients, that is, 12 out of 23 patients (Patients 4, 5, 8, 9, 10, 11, 12, 13, 16, 17, 19, and 23). This was in line with the approximately 60% success rate reported for exome capture strategies to identify Mendelian disease genes [4], but represented a 5-fold increase in mutation detection as compared to the APEX assay [6]. A solution-based targeted exon capture assay similar to IROme had also identified disease-causing mutations in 11 out of 17 families affected by various retinal degenerations (65%) [17]. In contrast, in a cohort of 100 RP patients, array-based targeted exon capture resulted in the identification of pathogenic mutations in 36 individuals (36%) [15]. Amplicon-based approaches identified potential mutations in 24% of patients affected by retinal degenerations (5/21) [18], in 79% of ADRP patients (15/19) [19], and 24% of LCA patients (4/17) [20].

In addition to the control (patient 5), only the p.PROM1-R373C mutation identified in patient 10 had been previously described [21], further underscoring the importance of screening RP-linked genes for the presence of new mutations.

The workflow for variant detection was not immediately successful for two patients. For patient 9, a deletion of exons 45–47 in *ABCA4* was only found by analyzing the coverage data. For patient 16, the 33 bp insertion in *PRPF31* was detected by Sequence Pilot, but not Reference Mapper software.

Potential mutations were found in three patients (13%). Patient 1 inherited from her healthy mother a heterozygous p.C2ORF71-R571delRTVVPP mutation and from her healthy father a heterozygous p.FSCN2-P231S mutation. Digenic RP has been linked so far to heterozygous PRPH2 and ROM1 mutations [2], and further analyses will be necessary to validate this molecular diagnostic. Patient 2 and 20 had, respectively, two and one potential mutation, but no family members were available to confirm the result.

Results were questionable for two additional patients. Patient 6 carried a p.RHO-R252P mutation that had been previously reported [22]. However, unaffected family members were not available to confirm this dominant mutation. Also, a heterozygous p.CRX-Q105X sequence variant was detected in patient 14, but his healthy mother was also carrying it.

Finally, no molecular diagnostic could be established for six patients (26%): in patients 18 and 21 no potential mutations were found by IROme analysis, in patients 7 and 15 the potential mutation did not segregate with disease in the family, and in patients 3 and 22 heterozygous mutations were found in genes only reported for recessive inheritance (*CLRN1, EYS*).

Of note, all these patients carry novel sequence variants in noncoding regions. To prioritize for potential disease-causing sequence variants in these regions, systematic annotation should not only cover splicing sites, 5′- and 3′-UTRs, but also implement detailed information about transcription factor binding sites and regulatory elements located in the potential proximal promoter regions. Promoter sequence variants could then be tested by reporter transactivation assays (e.g., luciferase reporter assays), but this time-consuming approach cannot be implemented in a routine molecular diagnostic lab.

## 4. Conclusions

The custom designed in solution-based targeted exon capture assay IROme efficiently detected disease-causing mutations in 55% of RP patients (12/23). A 99.7% coverage of the targeted regions was obtained. The first translated exon often contains sequences with a high GC content in its 5′-UTR that hinders an efficient capture [23]. Remarkably, more than 95% of exons 1 (60/63) were successfully enriched by IROme. In comparison, a pilot study carried out in our laboratory on 25 patients using whole exome sequencing (SureSelect, Agilent) resulted in no coverage of promoter regions, highly variable coverage of 3′-UTRs, and several genes had their first translated exon very poorly covered. For instance, the first exons of the following RP-linked genes could not be correctly analyzed: *C2ORF71*, *CA4*, *CABP4*, *CERKL*, *CNGA1*, *FAM161A*, *FSCN2*, *GUCY2D*, *IMPDH1*, *LPCAT1*, *MERTK*, *RDH12*, *RP9,* and *RPGR *(D. F. Schorderet, unpublished results). It is tempting to speculate that the additional sequences upstream of exon 1 included in IROme further enhanced the performance of the NimbleGen exome capture technology, that reportedly has more specific targeting and a higher percentage of on-target reads than competing products [23, 24]. However, because the costs for whole exome sequencing have dramatically decreased to about 1000 $ per patient, this method may in the future replace target enrichment and resequencing, providing that a new line of “whole exome” kits covering effectively all exons, including the first one, of all genes, will become commercially available [24].

Meanwhile, custom-designed target enrichment and subsequent next-generation sequencing are a cost-efficient approach for the molecular diagnosis of retinal dystrophies, also with respect to the relative ease of data handling and analysis [25]. Finally, the median global coverage of 17-fold observed with the IROme assay also indicated the possibility to include additional retinal degeneration-linked genes, newly discovered ones or candidate genes.

## Figures and Tables

**Table 1:** List of genes enriched by targeted sequence capture (IROme).

Gene	Alias	Chr	Chr location	Exons	Pathology
*ABCA4 *	RP19, STGD1, CORD3, and ARMD2	1	94458391-94586688 (rs)	50	ADRP, ARRP, ARCRD, and ARMD
*AIPL1 *	LCA4	17	6327057-6338519 (rs)	6	ARLCA, ADCRD
*BEST1 *	RP50, BMD, and VMD2	11	61717293-61732987 (fs)	11	ADRP, ARRP, and ADMD
*C2ORF71 *	RP54	2	29284556-29297127 (rs)	2	ARRP
*CA4 *	RP17	17	58227302-58236902 (fs)	8	ADRP
*CABP4 *	CSNB2B	11	67219877-67226699 (fs)	7	ARLCA, ARCSNB
*CEP290 *	LCA10, BBS14, and NPHP6	12	88442794-88535993 (rs)	53	ARLCA, ARBBS
*CERKL *	RP26	2	182401403-182545392 (rs)	14	ARRP, ARCRD
*CLRN1 *	RP61, USH3A	3	150643950-150690786 (rs)	3	ARRP
*CNGA1 *	RP49	4	47937994-48018689 (rs)	13	ARRP
*CNGA2 *		X	150906923-150913776 (fs)	6	
*CNGB1 *	RP45	16	57917847-58005020 (rs)	33	ARRP
*CRB1 *	LCA8, RP12	1	197170592-197447585 (fs)	12	ARRP, ARLCA
*CRX *	LCA7, CORD2	19	48325097-48364769 (fs)	4	ADRP, ADLCA, ARLCA, and ADCRD
*DHDDS *	RP59	1	26758773-26797785 (fs)	9	ARRP
*EYS *	RP25	6	64429876-66417118 (rs)	43	ARRP
*FAM161A *	RP28	2	62051989-62081278 (rs)	6	ARRP
*FSCN2 *	RP30	17	79495422-79504156 (fs)	5	ADRP, ADMD
*GUCA1B *	RP48, GCAP2	6	42152139-42162694 (rs)	4	ADRP, ADMD
*GUCY2D *	LCA1, CORD6	17	7905988-7923658 (fs)	20	ARLCA, ADCRD
*IMPDH1 *	LCA11, RP10	7	128032331-128050306 (rs)	17	ADRP, ADLCA
*IMPG2 *	RP56, sparcan	3	100945570-101039404 (rs)	20	ARRP
*IQCB1 *	NPHP5	3	121488610-121553926 (rs)	15	ARLCA
*KLHL7 *	RP42	7	23145353-23215040 (fs)	12	ADRP
*LCA5 *	Lebercilin	6	80194708-80247175 (rs)	8	ARLCA
*LPCAT1 *	AYTL2	5	1456595-1524092 (rs)	14	ARLCA
*LRAT *	LCA14	4	155548097-155674270 (fs)	4	ARRP, ARLCA
*MERTK *	RP38	2	112656056-112787138 (fs)	19	ARRP
*NR2E3 *	RP37, PNR	15	72084977-72110559 (fs)	8	ADRP, ARRP, and ARESCS
*NRL *	RP27	14	24549316-24584223 (rs)	3	ADRP, ARRP, and ARESCS
*OFD1 *	RP23	X	13752832-13787480 (fs)	23	XRP
*OTX2 *		14	57267426-57277197 (rs)	5	ADLCA
*PDE6A *	RP43	5	149237519-149324356 (rs)	22	ARRP
*PDE6B *	RP40, CSNBAD2	4	619373-664571 (fs)	23	ARRP, ADCSNB
*PDE6G *	RP57	17	79617489-79623607 (rs)	4	ARRP
*PRCD *	RP36	17	74523871-74541458 (fs)	5	ARRP
*PROM1 *	RP41, STGD4, CORD12, and MCDR2	4	15964699-16086001 (rs)	28	ARRP, ADCRD, and ADMD
*PRPF3 *	RP18	1	150293925-150325671 (fs)	16	ADRP
*PRPF6 *	RP60	20	62612488-62664453 (fs)	21	ADRP
*PRPF8 *	RP13	17	1553923-1588154 (rs)	43	ADRP
*PRPF31 *	RP11	19	54618837-54635140 (fs)	14	ADRP
*PRPH2 *	RDS, RP7	6	42664340-42690312 (rs)	3	ADRP, ADMD, ADCRD, and digenic
*RBP3 *	IRBP	10	48381487-48390991 (rs)	4	ARRP
*RDH12 *	LCA13, RP53	14	68168603-68201169 (fs)	8	ADRP, ARLCA
*RGR *	RP44	10	86004809-86019716 (fs)	7	ADRP, ARRP, and ADCA
*RHO *	RP4, CSNBAD1	3	129247483-129254012 (fs)	5	ADRP, ARRP, and ADCSNB
*RLBP1 *	CRALBP	15	89753098-89764922 (rs)	9	ARRP
*ROM1 *		11	62379194-62382592 (fs)	3	ADRP, digenic
*RP1 *		8	55471729-55682531 (fs)	4	ADRP, ARRP
*RP2 *		X	46696375-46741793 (fs)	5	XRP
*RP9 *	PAP1	7	33134409-33149013 (rs)	7	ADRP
*RPE65 *	LCA2, RP20	1	68894505-68915642 (rs)	14	ARRP, ARLCA
*RPGR *	RP3, CORDX1	X	38128424-38186817 (rs)	19	XRP, XCRD, XMD
*RPGRIP1 *	LCA6, CORD13	14	21756098-21819460 (fs)	24	ARLCA, ARCRD
*SAG *	RP47, Arrestin	2	234216309-234255701 (fs)	16	ARRP, ARCSNB
*SEMA4A *	RP35, CORD10	1	156117157-156147543 (fs)	16	ADRP, ARRP, and ADCRD
*SNRNP200 *	RP33	2	96940074-96971297 (rs)	45	ADRP
*SPATA7 *	LCA3	14	88851268-88936694 (fs)	12	ARLCA
*TOPORS *	RP31	9	32540542-32552551 (rs)	3	ADRP
*TUB *		11	8040791-8127659 (fs)	13	
*TULP1 *	LCA15, RP14	6	35465651-35480715 (rs)	15	ARRP, ARLCA
*USH2A *	RP39	1	215796236-216596738 (rs)	73	ARRP
*ZNF513 *	RP58	2	27600098-27603657 (rs)	4	ARRP

Genes are listed alphabetically according to their official gene symbol, and, in addition, gene aliases commonly used in ophthalmic research provided. Chromosomal (chr) location is based on the Homo sapiens high-coverage assembly GRCh37, yielding in the UCSC hg19 database (fs: forward strand; rs: reverse strand). For each gene the number of exons is listed. Targeted sequence capture was directed against genes causing autosomal dominant (AD), autosomal recessive (AR) X-linked (X), retinitis pigmentosa (RP), and Leber congenital amaurosis (LCA). Other retinopathies caused by a given gene are also indicated: cone or cone-rod dystrophy (CRD), macular degeneration (MD), congenital stationary night blindness (CSNB), Bardet-Biedl syndrome (BBS), enhanced S-cone syndrome (ARESCS), and chorioretinal atrophy (CA). Heterozygote ROM1 and PRPH2 mutations cause digenic disease. ORF15 of RPGR was not included in the assay.

**Table 2:** Validation of IROme by APEX.

Pat number	nt tested by APEX	nt detected by IROme	Mean cvg	% reads homo
1	557	100%	25	98.9
2	558	100%	26	99.4
3	558	100%	22	99.2
4	547	100%	20	98.3

The nucleotides (nt) tested by APEX represent validated RP-linked mutations or variants. The mean coverage (cvg) refers to the average of the coverage of all exons where the mutations are located. The percentage of sequence reads generated by IROme and correctly calling the nucleotides at homozygous state are indicated.

**Table 3:** Synopsis of molecular diagnostic on RP patients by IROme.

Pat number	Total seq Mb	Read length bp	Median fold cvg	Total seq var	cds seq var	filt. seq var	prio. seq var	Test/val seq var	Potential mutation	cvg pot mut	mut reads %	Cosegregate family
1	47	453	21.3	1206	114	51	8	2/2	p.C2ORF71-R571_P576del p.FSCN2-P231S	38 25	55.3 44	M het norm F het norm
2	47.6	433	20.9	1217	98	44	7	2/2	p.PDE6B-H337R p.OTX2-G222R	21 52	100 48	? ?
3	42.2	416	17.0	1085	78	39	6	1/1	p.CLRN1-P134L	19	68.4	?
4	44.6	395	21.6	1173	95	42	5	3/3	p.RHO-Y191C	39	38.5	yes
5	24.4	429	13.8	894	104	45	1	1/1	p.TULP1-F529_A530dup	6	100	yes
6	31.7	422	16.2	1039	85	47	1	1/1	p.RHO-R252P	22	54.5	?
7	20.1	281	13.3	789	77	38	4	2/2	p.SAG-E11K p.IMPG2-G684R	30 34	56.7 38.2	no no
8	13.9	445	9.1	736	70	33	2	1/1	p.RP2-D161Y	22	45.5	yes
9	29	297	17.3	832	80	39	9	1/1	g.ABCA4-ex45-47del	0	0	yes
10	37.3	443	16.7	1247	93	46	3	3/3	p.PROM1-R373C	32	50	yes
11	50.2	440	21.5	1151	92	46	2	1/1	p.RP2-E20X	28	67.8	yes
12	49.1	394	23.8	1116	94	39	9	4/4	p.CNGB1-R765C	30	100	yes
13	33	436	14.6	1017	85	33	3	2/2	p.GUCY2D-V887G	18	94.4	yes
14	32.6	443	15.3	1205	93	42	3	1/1	p.CRX-Q105X	17	58.8	M het norm
15	32.7	442	14.2	1026	86	40	3	1/1	p.USH2A-P2630R	25	40	no
16	69.4	434	28.4	1246	87	41	1	1/1	p.PRPF31-E183_ins33bp	74	40	yes
17	16.7	452	9.8	861	82	43	2	2/2	p.PRPH2-L39P	18	50	yes
18	39.6	429	17.2	1826	85	35	3	1/1				
19	66.5	449	26.4	1298	103	45	5	2/2	p.PRPH2-S217_dup16bp	71	39.4	yes
20	47.1	358	19.8	1171	91	47	3	2/1	p.C2ORF71-L889P	23	39.1	?
21	47	363	17.3	1197	102	48	3	1/0				
22	36.6	354	14.7	1072	86	45	2	2/1	p.EYS-D2930G	38	60.5	?
23	53.3	393	22.4	1164	92	40	7	5/5	p.PRPF8-E2331X	38	44.7	yes

For each patient, the total number of Mb (10^6^ bp) sequenced on the Roche 454 GS Junior (total seq Mb) and the average read length (read length bp) are indicated. The median fold coverage (cvg) was extracted from the unique depth information. From all the sequence variants (total seq var), first only the sequence variants located in coding sequences were analyzed (cds seq var), with filtering (filt seq var) and prioritizing (prio seq var) according to Figure 2. The sequence variants eventually tested and validated by Sanger sequencing (test/val seq var) are also indicated. For each potential mutation, the coverage (cvg pot mut) and the percentage of sequence reads reporting the potential mutation (mut reads %) are indicated. For cosegregation analysis, “?” indicates absence of available family members and/or simplex cases. For patients 1 and 14, the mother (M) and/or the father (F) are healthy heterozygous carriers (het norm).

## References

[1] Hartong DT, Berson EL, Dryja TP (2006). Retinitis pigmentosa. *The Lancet*.

[2] Berger W, Kloeckener-Gruissem B, Neidhardt J (2010). The molecular basis of human retinal and vitreoretinal diseases. *Progress in Retinal and Eye Research*.

[3] Ferrari S, Di Iorio E, Barbaro V, Ponzin D, Sorrentino FS, Parmeggiani F (2011). Retinitis pigmentosa: genes and disease mechanisms. *Current Genomics*.

[4] Gilissen C, Hoischen A, Brunner HG, Veltman JA (2012). Disease gene identification strategies for exome sequencing. *European Journal of Human Genetics*.

[5] Zernant J, Külm M, Dharmaraj S (2005). Genotyping microarray (disease chip) for leber congenital amaurosis: detection of modifier alleles. *Investigative Ophthalmology and Visual Science*.

[6] Ávila-Fernández A, Cantalapiedra D, Aller E (2010). Mutation analysis of 272 Spanish families affected by autosomal recessive retinitis pigmentosa using a genotyping microarray. *Molecular Vision*.

[7] Metzker ML (2010). Sequencing technologies—the next generation. *Nature Reviews Genetics*.

[8] Biesecker LG (2010). Exome sequencing makes medical genomics a reality. *Nature Genetics*.

[9] Ng SB, Turner EH, Robertson PD (2009). Targeted capture and massively parallel sequencing of 12 human exomes. *Nature*.

[10] Ng SB, Buckingham KJ, Lee C (2010). Exome sequencing identifies the cause of a mendelian disorder. *Nature Genetics*.

[11] Bamshad MJ, Ng SB, Bigham AW (2011). Exome sequencing as a tool for Mendelian disease gene discovery. *Nature Reviews Genetics*.

[12] Mamanova L, Coffey AJ, Scott CE (2010). Target-enrichment strategies for next-generation sequencing. *Nature Methods*.

[13] Loman NJ, Misra RV, Dallman TJ (2012). Performance comparison of benchtop high-throughput sequencing platforms. *Nature Biotechnology*.

[14] Mataftsi A, Schorderet DF, Chachoua L (2007). Novel TULP1 mutation causing leber congenital amaurosis or early onset retinal degeneration. *Investigative Ophthalmology and Visual Science*.

[15] Neveling K, Collin RW, Gilissen C (2012). Next-generation genetic testing for retinitis pigmentosa. *Human Mutation*.

[16] Vandenbroucke I, Van Marck H, Verhasselt P (2011). Minor variant detection in amplicons using 454 massive parallel pyrosequencing: experiences and considerations for successful applications. *Biotechniques*.

[17] Audo I, Bujakowska KM, Leveillard T (2012). Development and application of a next-generation-sequencing (NGS) approach to detect known and novel gene defects underlying retinal diseases. *Orphanet Journal of Rare Diseases*.

[18] Bowne SJ, Sullivan LS, Koboldt DC (2011). Identification of disease-causing mutations in autosomal dominant retinitis pigmentosa (adRP) using next-generation DNA sequencing. *Investigative Ophthalmology and Visual Science*.

[19] Song J, Smaoui N, Ayyagari R (2011). High-throughput retina-array for screening 93 genes involved in inherited retinal dystrophy. *Investigative Ophthalmology and Visual Science*.

[20] Coppieters F, de Wilde B, Lefever S (2012). Massively parallel sequencing for early molecular diagnosis in Leber congenital amaurosis. *Genetics in Medicine*.

[21] Yang Z, Chen Y, Lillo C (2008). Mutant prominin 1 found in patients with macular degeneration disrupts photoreceptor disk morphogenesis in mice. *Journal of Clinical Investigation*.

[22] Grinberg ER, Dzhemileva LI, Khusnutdinova EK (2007). The novel R252P Mutation of the RHO gene in patients with retinitis pigmentosa from Bashkortostan. *Molecular Biology*.

[23] Frommolt P, Abdallah AT, Altmüller J (2012). Assessing the enrichment performance in targeted resequencing experiments. *Human Mutation*.

[24] Sulonen AM, Ellonen P, Almusa H (2011). Comparison of solution-based exome capture methods for next generation sequencing. *Genome Biology*.

[25] Fromer M, Moran JL, Chambert K (2012). nd statistical genotyping of copy-number variation from whole-exome sequencing depth. *The American Journal of Human Genetics*.

